# Excess influenza hospital admissions and costs due to the 2009 H1N1 pandemic in England

**DOI:** 10.1002/hec.3834

**Published:** 2018-10-18

**Authors:** Krystal Lau, Katharina Hauck, Marisa Miraldo

**Affiliations:** ^1^ Department of Management Imperial College Business School London UK; ^2^ Centre for Health Economics & Policy Innovation (CHEPI) Imperial College Business School London UK; ^3^ Department of Infectious Disease Epidemiology, School of Public Health Imperial College London London UK

**Keywords:** cost, H1N1 pandemic, hospital admissions, SARIMA, time series

## Abstract

Influenza pandemics considerably burden affected health systems due to surges in inpatient admissions and associated costs. Previous studies underestimate or overestimate 2009/2010 influenza A/H1N1 pandemic hospital admissions and costs. We robustly estimate overall and age‐specific weekly H1N1 admissions and costs between June 2009 and March 2011 across 170 English hospitals. We calculate H1N1 admissions and costs as the difference between our administrative data of all influenza‐like‐illness patients (seasonal and pandemic alike) and a counterfactual of expected weekly seasonal influenza admissions and costs established using time‐series models on prepandemic (2004–2008) data.

We find two waves of H1N1 admissions: one pandemic wave (June 2009–March 2010) with 10,348 admissions costing £20.5 million and one postpandemic wave (November 2010–March 2011) with 11,775 admissions costing £24.8 million. Patients aged 0–4 years old have the highest H1N1 admission rate, and 25‐ to 44‐ and 65+‐year‐olds have the highest costs. Our estimates are up to 4.3 times higher than previous reports, suggesting that the pandemic's burden on hospitals was formerly underassessed. Our findings can help hospitals manage unexpected surges in admissions and resource use due to pandemics.

## 1 INTRODUCTION

Pandemics severely threaten health systems due to difficulty in forecasting number and severity of cases, unavailability of immediate vaccines, and insufficient antiviral stockpiles to meet demand (Centers for Disease Control and Prevention & National Center for Immunization and Respiratory Diseases, 2016). An influenza pandemic could cost approximately $500 billion worldwide, making preparedness imperative (Fan, Jamison, & Summers, 2018). During the 2009 influenza A/H1N1 pandemic, concentrated waves of infection led to surges in secondary care demand (Department of Health Pandemic Influenza Preparedness Team, 2011) and associated costs in the United States (Ampofo et al., 2006), United Kingdom (Campbell et al., 2011; Mytton, Rutter, & Donaldson, 2012), Greece (Zarogoulidis et al., 2012), New Zealand (Wilson et al., 2012), Spain (Galante et al., 2012), and Australia (Higgins et al., 2011). Apart from admission surges, influenza affects healthcare workers themselves (Department of Health Pandemic Influenza Preparedness Team, 2011), resulting in hospital capacity pressures due to sickness‐related absences. Lastly, work absences throughout all sectors can lead to severe productivity and economic losses (Grosse, 2009). The Institute of Medicine Board on Global Health Forum's on Microbial Threats (2010) advises that secondary care capacity planning is key for future pandemic preparation; although the 2009 H1N1 pandemic was milder than expected, it pushed admission numbers near many hospitals' capacities. Hospitals may have difficulty coping with admission surges during future, more severe, influenza pandemics due to lack of staff, antiviral treatment, and ventilation equipment (Institute of Medicine Board on Global Health Forum on Microbial Threats, 2010).

The objective of our study is to provide robust estimates of the number and cost of H1N1 admissions in excess to seasonal influenza hospitalizations during the pandemic and a postpandemic influenza season in England. We employ time‐series methodologies on data sets of all influenza admissions (*n* = 29,403) and their costs to National Health Service (NHS) hospitals in England between 2004 and 2011.

Although it is commonly argued that pandemics are extremely costly to health systems, considerable ambiguity exists surrounding the extent to which H1N1 burdened hospitals. Extant literature provides varying estimates of weekly H1N1 hospital admission rates (0.11 [Campbell et al., 2011]–1.72 [Shrestha et al., 2011] per 100,000 population) and unit costs ($4,086.04 [Zarogoulidis et al., 2012]–$11,646.18 [Wilson et al., 2012]) during the pandemic and postpandemic periods (Tables S1 and S2). Although differences in countries' health systems may partly explain these variations, substantial data and method discrepancies between these studies may also bias estimates. The symptoms of pandemic and nonpandemic influenza are difficult to distinguish, and H1N1 cases are often undocumented in patient records. Therefore, studies face the problem of identifying influenza‐like‐illness (ILI) admissions that are due to H1N1. Some studies' data only count laboratory‐confirmed H1N1 cases (Bolotin et al., 2012; Campbell et al., 2011; Helferty et al., 2010; McLean & Pebody, 2010; Shrestha et al., 2011), but then, risk downward biased estimates because they miss undiagnosed H1N1 patients (Reed et al., 2009). Further, some studies' data come from a fraction of all hospitals in a health system (Bolotin et al., 2012; Campbell et al., 2011; McLean & Pebody, 2010; Shrestha et al., 2011), which may be an unrepresentative sample. Bolotin et al. (2012) exclude two of 10 English regions (North East England and West Midlands), which may systematically differ from the remaining eight regions. Campbell et al. (2011) and McLean and Pebody (2010) exclude 23% of U.K. hospitals; although the missing data does not differ from included hospitals on number of facilities and beds, it may nonrandomly differ on other factors such as age distribution and patient severity. These can impact estimates of admission numbers and costs. Although Shrestha et al. (2011) correct for underreporting, they use a nonrandomly distributed group of hospitals that covers only 7% of the U.S. population. Only Helferty et al. (2010) use laboratory‐confirmed hospitalizations from all 13 Canadian provinces and territories. Other studies use Bayesian modeling on multiple laboratory‐confirmed data sets to help overcome ascertainment bias (Presanis et al., 2009, 2011). However, they assume equal probabilities of H1N1 testing by physicians across age groups despite studies showing that physicians are less likely to order influenza tests for elderly patients than younger ones (Hartman, Zhu, Edwards, Griffin, & Talbot, 2018). Thus, the detection probability for the elderly is likely lower than for others. Mytton et al. (2012) avoid data‐related biases by counting all ILI admissions from an administrative hospitalization data set but fail to distinguish H1N1 from nonpandemic ILI patients, which likely biases their estimates upwards.

Moreover, while studies agree that children experienced the highest H1N1 admission rates (Bolotin et al., 2012; Campbell et al., 2011; Helferty et al., 2010; McLean & Pebody, 2010; Shrestha et al., 2011; Van't Klooster et al., 2010), their weekly estimates widely vary, between 0.06 (Bolotin et al., 2012) and 3.31 (Helferty et al., 2010) per 100,000 population (Table S1). Again, this could be due to demographic and health system differences between countries, but inconsistent weekly admission rate estimates persist between English studies (0.66 [Campbell et al., 2011] and 0.06 [Bolotin et al., 2012]).

Finally, pandemic costing studies suffer from restricted generalizability due to small sample size (Galante et al., 2012; Higgins et al., 2011; Wilson et al., 2012). Relying on data from fewer than four hospitals impedes generalizability of costing differences between providers to the whole health system. Uscher‐Pines and Elixhauser (2013) analyze a stratified sample of 18.87% of U.S. hospitals but calculate unit cost using all influenza, not exclusively H1N1, inpatient stays. As pandemic influenza often requires more extensive and expensive care than seasonal influenza, the inclusion of seasonal influenza costs is likely to bias H1N1 unit cost estimates downward.

We contribute to the literature by using seasonal autoregressive integrated moving average (SARIMA) models to analyze the universe of patient‐level admissions and costs from 170 public hospitals in England from 2004 to 2011. To our knowledge, this is the first application of time‐series methods to estimate total and age‐specific excess ILI hospital admissions and costs attributable to H1N1 using administrative data, allowing us to substantially improve over existing studies' data and methods. Our study is the first to estimate unit costs based on patient‐level data using all public hospitals within a health system. We compare our findings with other countries' studies to determine the magnitude of bias in their estimates. Our results will help decision makers improve their admission surge response plans for future influenza pandemics.

The remainder of this paper is organized as follows: Section 2 describes the data, Section 3 demonstrates the econometric method, Section 4 outlines results, and Section 5 offers discussion and concluding remarks.

## 2 DATA

We use Hospital Episode Statistics (HES; National Health Service Digital, 2016), an administrative hospital records data set containing information on the universe of inpatient admissions (*n* = 132,532,270), including age and primary and secondary diagnoses coded according to the International Statistical Classification of Diseases and Related Health Problems, 10th revision (ICD‐10), across all NHS hospitals in England between April 2004 and March 2011. HES is preferred over laboratory‐confirmed data as the latter may not capture a significant proportion of H1N1 patients. Physicians may not have tested for H1N1 because of time constraints, certainty of accurate diagnosis, or they deemed it redundant since H1N1 would warrant similar treatment as other severe influenza. We extract all ILI inpatients with laboratory‐confirmed and unconfirmed H1N1, seasonal and other influenzas, as their primary or secondary diagnosis (ICD‐10 codes J09—certain identified influenza, J10—seasonal influenza, and J11—unidentified influenza) following Pebody et al. (2010). This approach captures ILI patients missed in laboratory‐confirmed data sets and those with comorbidities aggravated by pandemic influenza who may have been admitted under a comorbidity primary diagnosis and an influenza secondary diagnosis. Including these complex patients is important as they may require more costly treatment because of coinfections with influenza and thus contribute to the pandemic's true impact on hospital admissions and costs. We identify 29,403 ILI admissions across 170 hospitals and collapse by week to generate a time series of 416 weekly ILI admissions.

We calculate the cost of each ILI admission per hospital using the Healthcare Resource Groups (HRGs), developed by the Department of Health Payment by Results (PbR) Team (2012). Every HES inpatient admission is assigned an HRG, which aggregates patients with medically comparable diagnoses and procedures that utilize similar amounts of resources (National Health Service England, 2016). Each HRG is linked to a reference cost unique to the hospital and admission type (day case, elective, or nonelective), which is the patient's true cost at a given hospital. Nationwide admission type and HRG‐specific base tariffs are calculated as the mean of reference costs lagged 3 years collected from all NHS hospitals. HRG‐specific daily excess bed‐day payments are added to the base tariff for each day that a patient stays beyond the expected length of stay or trim point, calculated by the PbR team. This is then multiplied by a market forces factor index, also calculated by the PbR team, which estimates unavoidable cost differences between hospitals due to location, such as labor, land, and building costs. The final tariff represents the ultimate cost to the commissioner. All costs are inflated to March 2011 values using the consumer price index (Office for National Statistics, 2017b).

Tariffs are first matched with patients according to HRG and admission type. Remaining unmatched admissions are matched only by HRG using an unweighted average of each HRG's tariffs across admission types. Of the 29,403 eligible HES admissions, 2.6% of ILI admissions could not be matched to tariffs in any manner and are excluded from our analysis. Differences in gender between matched (*M* = 0.54, *SD* = 0.003) and unmatched (*M* = 0.53, *SD* = 0.02) admissions (*Z* = 0.59, *p* = 0.55) are not statistically significant. Matched admissions are significantly younger (*M* = 32.54, *SD* = 0.14) than unmatched (*M* = 17.58, *SD* = 3.52), *t*(29,906) = 4.78, *p* < 0.001, and have shorter lengths of stay (*M* = 6.84, *SD* = 0.09) than unmatched admissions (*M* = 17.58, *SD* = 3.52), *t*(30,154) = 13.15, *p* < 0.001. Although this is a small portion of our data set, our cost estimates may be conservative. The final costing data set includes 28,626 admission–tariff matches across NHS hospitals in England between April 2004 and March 2011 to produce a time series of 416 observations of weekly costs of ILI admissions.

For our subsample analysis by age group, we consider 4,992 observations over Bolotin et al.'s (2012) age bands: 0–4, 5–14, 15–24, 25–44, 45–64, 65+ years.

## 3 METHODS

### 3.1 SARIMA models

We identify excess ILI admissions and costs by fitting multiplicative SARIMA models (Box, Jenkins, Reinsel, & Ljung, 2015) to our nonpandemic observations, April 2004–October 2008, to create a counterfactual of typical nonpandemic weekly ILI admission numbers and costs for the pandemic and postpandemic periods between November 2008 and March 2011. 1We use definite prepandemic data (April 2004 to October 2008) as our baseline to establish our counterfactual nonpandemic estimates (November 2008 to March 2011). The counterfactual enabled us to determine when the pandemic (June 2009 to March 2010) and postpandemic (November 2010 to March 2011) periods occurred within this time frame. We use SARIMA models to disentangle the weekly number and cost of excess ILI admissions due to the pandemic from the number and costs we would see in a typical, nonpandemic year. SARIMA, a multiplicative extension of autoregressive integrated moving average (ARIMA), models are used because our data exhibit seasonal winter peaks in ILI admissions. The general ARIMA (p, d, q) model is as follows:

(1) $${\left(1-B\right)}^{d}\;{\hat{Y}}_{t}=\alpha +\frac{\theta \left(B\right)}{\phi \left(B\right)}\;\varepsilon_{t},$$

where $\;{\hat{Y}}_{t}$ is a forecasted observation of *Y*, *B* is the backshift operator (*BY*_*t*_ = *Y*_*t* − 1_), *α* is a constant, *d* is the order of differencing, and *ε*_*t*_ is the random error. The regular autoregressive (AR) operator is *ϕ*(*B*), where *ϕ*(*B*) = 1 − ϕ_1_*B*−_⋯_ − ϕ_*p*_*B*^*p*^, with order *p*. The regular moving average (MA) operator is *θ*(*B*) where *θ*(*B*) = 1 − θ_1_*B*−_⋯_ − *θ*_*q*_*B*^*q*^, with order *q*.

SARIMA is written as ARIMA (p, d, q; P, D, Q)_S_ and expressed as follows:

(2) $${\left(1-B\right)}^{d}\;{\left(1-B^{s}\right)}^{D}\;{\hat{Y}}_{t}=\alpha +\frac{\theta \left(B\right)\;\theta_{s}\left(B^{s}\right)}{\phi \left(B\right)\;\phi_{s}\left(B^{s}\right)}\;\varepsilon_{t},$$

where *S* is the number of periods per season and *D* is the seasonal order of differencing. The seasonal AR operator is *ϕ*_*s*_(*B*^*s*^), where *ϕ*_*s*_(*B*^*s*^) = 1 − ϕ_*s*,1_*B*^*s*^−_⋯_ − ϕ_*s*,*P*_ $B^{s^{P}}$, with order P. The seasonal MA operator is *θ*_*s*_(*B*^*s*^), expressed as *θ*_*s*_(*B*^*s*^) = 1 − θ_*s*,1_*B*^*s*^−_⋯_ − θ_*s*,*Q*_ $B^{s^{Q}}$, with order *Q*.

Following Box et al.'s (2015) model building procedure, we remove seasonality by setting *S* = 52 for our weekly data and generate a time series of the same week across prepandemic years to predict typical ILI admissions and costs that should occur in a nonpandemic year. We use an augmented Dickey–Fuller test for stationarity such that our time‐series' mean, variance, and autocorrelation are constant over time. We then determine AR and MA lags using autocorrelation function and partial autocorrelation function plots, respectively, and select the most parsimonious lags combination that derives the minimum Akaike information criterion and Bayesian information criterion. Fourth, goodness‐of‐fit is tested on the SARIMA regression's standardized residuals using the Ljung–Box portmanteau (Q) test of autocorrelation and the Bartlett test of variances to ensure independence and constant mean and variance over time.

Pandemic weeks are defined as two or more consecutive weeks during the pandemic and postpandemic periods when actual ILI admissions or costs exceed the upper bound of the 95% prediction interval around the SARIMA counterfactual. The residual balance of ILI admissions and costs, calculated as the difference between actual and counterfactual values, is considered excess beyond nonpandemic influenza and is attributed to H1N1.

Overall, we built and ran 28 SARIMA models (Tables S3–S5). For each pandemic and postpandemic period, one model is for overall admissions, six for each age group subcategorization for admissions, one for overall costs, and six for each age group subcategorization for costs, totaling 14 models for admissions and 14 models for costs. We specify ILI hospital admissions (*N*) SARIMA models as follows:

(3) $${\left(1-B\right)}^{d_{N}}\;{\left(1-B^{s}\right)}^{D_{N}}\;{\hat{Y}}_{\mathrm{Nt}}=\alpha +\frac{\theta {\left(B\right)}_{N}\;\theta_{s}{\left(B^{s}\right)}_{N}}{\phi {\left(B\right)}_{N}\;\phi_{s}{\left(B^{s}\right)}_{N}}\;\varepsilon_{t},$$

where ${\hat{Y}}_{\mathrm{Nt}}$ is the counterfactual of ILI admissions in week *t*.

The costs for ILI admissions (*C*) SARIMA models are specified as follows:

(4) $${\left(1-B\right)}^{d_{C}}\;{\left(1-B^{s}\right)}^{D_{C}}\;{\hat{Y}}_{\mathrm{Ct}}=\alpha +\frac{\theta {\left(B\right)}_{C}\;\theta_{s}{\left(B^{s}\right)}_{C}}{\phi {\left(B\right)}_{C}\;\phi_{s}{\left(B^{s}\right)}_{C}}\;\varepsilon_{t},$$

where ${\hat{Y}}_{\mathrm{Ct}}$ is the counterfactual of costs for ILI admissions in week *t*.

Overall and age group weekly excess admission rates per 100,000 are estimated by dividing excess ILI admissions by the population size of the given age group then multiplied by 100,000. Census data from the Office for National Statistics (2017a) provides population size by age group. Overall and age group weekly unit costs are calculated by dividing excess ILI costs by excess ILI admissions. One‐way analysis of variance and Bonferroni multiple comparison tests for significant differences between age groups and between the pandemic and postpandemic periods are performed. We conduct all statistical analyses using Stata/MP statistical software, version 14 (College Station, TX, USA).

### 3.2 Comparison to other countries

We perform back‐of‐the‐envelope calculations to improve other countries' cost estimates for H1N1 hospitalizations that may be underreported by previous studies. We identify studies estimating overall and/or age‐specific H1N1 admission volume based on the laboratory‐confirmed H1N1 hospitalization data and H1N1 unit costs in the United States, Spain, Greece, Australia, and New Zealand. We calculate an adjuster by dividing our SARIMA‐generated H1N1 admission rates by previous English studies' estimated weekly H1N1 admission rates (Bolotin et al., 2012; Campbell et al., 2011). We multiply other studies' H1N1 admission rate estimates by the adjuster to provide improved weekly H1N1 admission rates in each aforementioned country. These are multiplied by each country's reported unit cost to provide country‐specific H1N1 hospitalization cost estimates, representing improved assessments of H1N1's actual burden on these countries' hospitals.

## 4 RESULTS

### 4.1 Descriptive statistics

**Figure 1:**
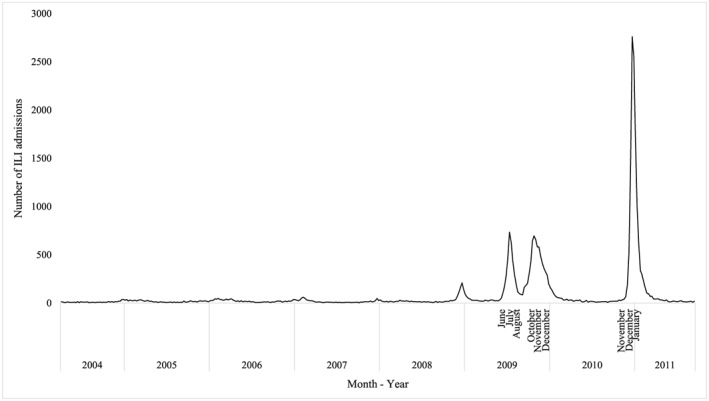
Weekly number of influenza‐like‐illness (ILI) admissions to 170 National Health Service (NHS) hospitals: April 2004–March 2011. Two peaks in ILI admissions during the pandemic period occurred in July and October 2009. One peak in ILI admissions during the postpandemic period occurred in December 2010

Our data of weekly total ILI admissions show small annual winter surges in the prepandemic years likely due to seasonal influenza. These are followed by distinct and large surges in ILI admissions in nonwinter months during the 2009 pandemic and postpandemic influenza season in 2010/2011 (Figure 1).

Patients aged 25–44 years old and 5–14 years old are the largest (26%) and smallest (8%) groups of total ILI hospital admissions, respectively (Table 1). Seasonal influenza (ICD‐code J10) is the most frequent diagnosis for all patients (58–78%). Most ILI patients younger than 15 years old are admitted with “upper respiratory tract disorders” (25–65%) during the prepandemic years and with “lower respiratory tract disorders without acute bronchiolitis” (19–44%) in the pandemic/postpandemic years. Most over 15 years old are admitted with “other viral illness” (18–55%) throughout the prepandemic years. During the pandemic/postpandemic, most 15–44‐ and 45+‐year‐old patients are admitted with “other viral illnesses” without (28–33%) and with critical care (37–40%), respectively.

**Table 1:** Descriptive statistics on total influenza‐like‐illness (ILI) hospital admissions, International Statistical Classification of Diseases and Related Health Problems, 10th revision (ICD‐10) code distribution, and most frequent Healthcare Resource Group (HRG) code

Age group	Total ILI hospital admissions (%) (*N* = 29,403)	ILI ICD‐10 code distribution (%)^a^		Most frequently assigned HRG code	
		J10 (*N* = 20,328)	J11 (*N* = 9,086)	Prepandemic (2004–2008)	Pandemic and postpandemic (2009–2011)
0–4 years	6,847 (19%)	4,407 (78%)	1,207 (22%)	Upper respiratory tract disorder	Other viral illness
5–14 years	2,869 (8%)	1,856 (76%)	579 (24%)	Upper respiratory tract disorder	Other viral illness
15–24 years	4,430 (13%)	2,615 (66%)	1,342 (34%)	Lower respiratory tract disorder	Other viral illness without critical care
25–44 years	9,241 (26%)	5,133 (66%)	2,649 (34%)	Lower respiratory tract disorder	Other viral illness with critical care
45–64 years	7,364 (20%)	4,175 (71%)	1,743 (29%)	Lower respiratory tract disorder	Other viral illness with critical care
65+ years	5,324 (14%)	2,412 (58%)	1,566 (42%)	Lower respiratory tract disorder	Other viral illness with critical care

a The total number ILI hospital admissions (*N* = 29,403) is less than the combined total of ILI admissions with ICD‐10 codes J10 and J11 (*N* = 20,328 + 9,086 = 29,414) because 11 patients have both diagnoses J10 and J11 and thus are counted twice. No ILI admissions with ICD‐10 code J09 were found in our data set.

### 4.2 Excess ILI admission numbers and costs

We estimate 22,123 ILI hospital admissions *exceeding* the SARIMA‐predicted number of ILI admissions based on a nonpandemic year and therefore ascribed to H1N1 across the entire pandemic/postpandemic period. Furthermore, the NHS spent an extra £45,381,756.03 throughout the pandemic/postpandemic period when compared with predicted ILI‐related costs based on the nonpandemic period. This excess cost is approximately 25% of the English NHS' 2010/2011 infectious disease program budget (Harker, 2012).

#### 4.2.1 SARIMA models—pandemic

**Figure 2:**
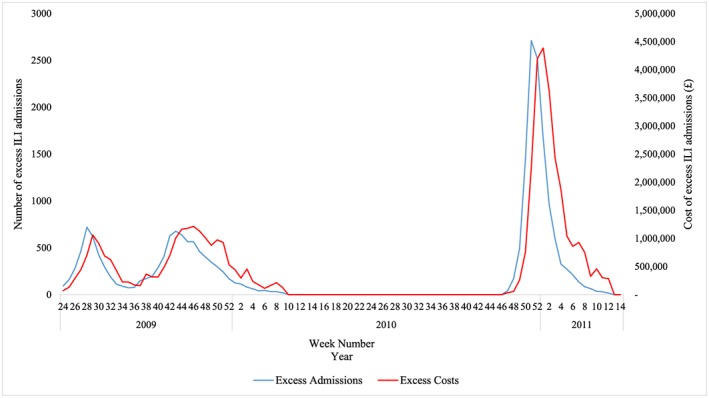
Excess weekly influenza‐like‐illness (ILI) hospital admissions (blue) and costs (red) during the pandemic and postpandemic influenza season calculated as the difference between actual and seasonal autoregressive integrated moving average (SARIMA)–estimated observations: June 2009–April 2011. We did not consider weeks 10–46 as pandemic weeks because ILI admissions were not significantly higher than in the prepandemic period's comparable weeks [Colour figure can be viewed at wileyonlinelibrary.com]

Our SARIMA models show two waves of excess ILI admissions during the pandemic, totaling a significant *excess* of 10,348 admissions above seasonal ILI and due to H1N1 (Figure 2). The pandemic also resulted in a significant excess of £20,563,000 in hospital costs over our predicted nonpandemic ILI costs (Figure 2).

We estimate an average excess admission rate of 0.70/100,000 and excess unit cost of £2,488 across all ages (Table 2). The 0‐ to 4‐year‐old age group has the highest weekly excess admission rate of 1.76/100,000, which is significantly higher than for all other age groups. Excess ILI admission rates decrease with age, as patients over the age of 65 years old (elderly) have the lowest weekly excess admission rate at 0.24/100,000. This suggests that incidence was lower among the elderly population, possibly due to them having preexisting levels of antibodies to combat the H1N1 virus (Centers for Disease Control and Prevention, 2009). However, the elderly has the highest average excess unit cost at £3,299 maybe because age‐related comorbidities increase the likelihood of influenza‐related complications. There is evidence that those with preexisting conditions had longer lengths of stays during the pandemic, which may explain their higher average unit cost (Campbell et al., 2011).

**Table 2:** Average excess influenza‐like‐illness (ILI) admission rates and unit costs (£) attributed to H1N1 during the 2009 pandemic and 2010/2011 postpandemic influenza season and ratios of excess admission rates and unit costs during the 2010/2011 postpandemic influenza season compared to the 2009 pandemic

	2009 pandemic (June 8, 2009, to March 7, 2010)		2010/2011 postpandemic influenza season (November 22, 2010 to March 27, 2011)			
Age group	Average excess admission rate due to H1N1 [95% CI]	Average excess unit cost (£) due to H1N1 [95% CI]	Average excess admission rate due to H1N1 [95% CI]	Average excess unit cost (£) due to H1N1 [95% CI]	Average excess admission rate ratio: postpandemic vs. pandemic [95% CI]	Average excess unit cost ratio: postpandemic vs. pandemic [95% CI]
0–4 years	1.76 [1.48, 2.04]	2,123.30 [483.01, 4,729.61]	3.23 [3.01, 3.45]	5,848.76 [3,585.87, 8,111.65]	1.83 [1.27, 2.64]	2.75 [2.73, 2.78]
5–14 years	0.66 [0.59, 0.72]	2,293.38 [526.28, 4,060.49]	0.53 [0.48, 0.59]	3,376.43 [640.73, 7,393.60]	0.81 [0.34, 1.76]	1.47 [1.46, 1.49]
15–24 years	0.64 [0.57, 0.71]	2,237.61 [1,980.47, 2,494.75]	1.43 [1.38, 1.49]	3,516.54 [2,798.46, 4,234.61]	1.93 [1.09, 3.42]	1.57 [1.56, 1.59]
25–44 years	0.48 [0.42, 0.54]	2,191.37 [1,785.63, 2,597.11]	1.30 [1.25, 1.35]	7,422.32 [6,392.79, 8,451.86]	2.72 [1.41, 5.32]	3.39 [3.36, 3.42]
45–64 years	0.35 [0.30, 0.39]	2,785.67 [2,191.01, 3,380.33]	1.16 [1.12, 1.20]	4,439.10 [4,046.54, 4,831.67]	3.36 [1.61, 7.29]	1.59 [1.58, 1.61]
65+ years	0.24 [0.15, 0.33]	3,299.10 [1,703.88, 4,894.31]	0.91 [0.84, 0.98]	4,140.99 [3,267.05, 5,014.92]	3.75 [1.57, 9.50]	1.26 [1.24, 1.27]
Total	0.70 [0.60, 0.81]	2,488.41 [1,445.05, 3,692.77]	1.43 [1.35, 1.51]	4,790.69 [3,455.24, 6,339.72]	2.03 [1.61, 2.54]	1.93 [1.92, 1.93]

#### 4.2.2 SARIMA models—postpandemic

The postpandemic influenza season has one, large wave (November 22, 2010–March 27, 2011) with an *excess* of 11,775 ILI admissions costing the NHS an excess of £24,819,000 in hospital costs (Figure 2) compared with the counterfactual of a nonpandemic period. We estimate an average excess admission rate of 1.43/100,000 and average excess unit cost of £4,791 (Table 2).

During the postpandemic period, patients across all age groups saw increases in their excess ILI admission rates compared with the pandemic except 5‐ to 14‐year‐olds, who had the lowest excess admission rate of 0.53/100,000 (Table 2). This rise is especially prominent for patients over the age of 25, whose average excess admission rate is 3.16 times their pandemic estimate. A few possible mechanisms can help explain this: Clinical studies suggest that an evolved H1N1 virus with an increased transmission rate and viral fitness could have led to increased admissions during the postpandemic period (Dorigatti, Cauchemez, & Ferguson, 2013). People may have also exhibited a self‐protection fatigue effect during the postpandemic period, whereby their perceived risk of infection lowered compared with the pandemic (Chen, Griffith, Cottrell, & Wong, 2013). This is consistent with a reduction in general practice consultations and decline in google searches for “flu” during the postpandemic period (Mytton et al., 2012).

Excess unit costs for ILI admissions are also higher across all age groups during the postpandemic period compared with the pandemic. Those aged 25–44 years old exhibit the highest excess unit cost of £7,422, which is 3.39 times their pandemic‐estimated unit cost. Furthermore, 0‐ to 4‐year‐olds have the second highest excess unit cost, £5,848.76, which is 2.8 times higher compared with the pandemic. Young adults used fewer antiviral medications at this time, which, in combination with an evolved H1N1 virus, could have resulted in more severe and expensive complications for otherwise healthy adults during the postpandemic period (Dorigatti et al., 2013; Mytton et al., 2012). An increase in severe bacterial infections across all age groups may be responsible for these higher excess unit costs (Zakikhany et al., 2011). A marked increase in bacterial coinfections with influenza among children and young adults may explain why these two age groups in particular experienced larger increases in excess unit costs (Zakikhany et al., 2011). Further age group comparisons are reported in Table 3.

**Table 3:** Bonferroni multiple comparison tests of excess influenza‐like illness (ILI) admission rates between age groups within and between the pandemic and postpandemic periods

			Mean difference (A–B)	
	Age group A	Age group B	Within pandemic and postpandemic period	Between pandemic and postpandemic period
Pandemic	0‐ to 4‐year‐olds	5‐ to 14‐year‐olds	1.10***	−170.08
		15‐ to 24‐year‐olds	1.12***	−114.31
		25‐ to 44‐year‐olds	1.28***	−68.07
		45‐ to 64‐year‐olds	1.41***	−662.37
		65+‐year‐olds	1.51***	−1,175.80
	5‐ to 14‐year‐olds	15‐ to 24‐year‐olds	0.02	55.77
		25‐ to 44‐year‐olds	0.18	102.01
		45‐ to 64‐year‐olds	0.32	−492.29
		65+‐year‐olds	0.41	−1,005.72*
	15‐ to 24‐year‐olds	25‐ to 44‐year‐olds	0.16	46.23
		45‐ to 64‐year‐olds	0.30	−548.06
		65+‐year‐olds	0.39	−1,061.49*
	25‐ to 44‐year‐olds	45‐ to 64‐year‐olds	0.14	−594.30
		65+‐year‐olds	0.23	−1,107.72**
	45‐ to 64‐year‐olds	65+‐year‐olds	0.10	−513.43
Postpandemic period	0‐ to 4‐year‐olds	5‐ to 14‐year‐olds	2.68***	2,472.33
		15‐ to 24‐year‐olds	1.96	2,332.23
		25‐ to 44‐year‐olds	1.91	−1,573.56
		45‐ to 64‐year‐olds	2.05	1,409.66
		65+‐year‐olds	2.31**	1,707.78
	5‐ to 14‐year‐olds	15‐ to 24‐year‐olds	−0.72	−140.10
		25‐ to 44‐year‐olds	−0.77	−4,045.89
		45‐ to 64‐year‐olds	−0.63	−1,062.67
		65+‐year‐olds	−0.37	−764.55
	15‐ to 24‐year‐olds	25‐ to 44‐year‐olds	−0.05	−3,905.79
		45‐ to 64‐year‐olds	0.09	−922.57
		65+‐year‐olds	0.35	−624.45
	25‐ to 44‐year‐olds	45‐ to 64‐year‐olds	0.14	2,983.22
		65+‐year‐olds	0.39	3,281.34
	45‐ to 64‐year‐olds	65+‐year‐olds	0.25	298.12

***p < 0.01. **p < 0.05. *p < 0.10.

### 4.3 Comparison to other countries

Our SARIMA‐estimated weekly H1N1 hospitalization rates—0.52 and 1.23 for the pandemic and postpandemic periods, respectively—are 1.2 (Mytton et al., 2012) to 4.7 (Campbell et al., 2011) times higher than those from most pandemic studies and 1.4 (Bolotin et al., 2012) to 1.6 (Mytton et al., 2012) times higher than those from postpandemic studies in England (Table S1). Papers with low estimates relied on laboratory‐confirmed cases from a small proportion of all hospitals in a country, with Campbell et al. (2011) using data from 77% and Bolotin et al. (2012) from 13.7% of all hospitals, which likely led them to underestimate the true number of H1N1 admissions. Even Mytton et al. (2012), who use the universe of influenza inpatient data in England, still provide conservative estimates for weekly H1N1 admission rates—0.43 for the pandemic and 0.79 for the postpandemic period, likely because they exclude some ILI diagnosis codes and therefore miss some H1N1 patients. By using SARIMA models on all hospital admissions within the NHS to disentangle typical ILI from excess admissions, we identify those ILI admissions that would not normally occur in a nonpandemic year but probably happened because of the pandemic and/or complications from H1N1 infections. Our results are confirmed by Presanis et al. (2011), who find a similar weekly H1N1 hospitalization rate—0.52—for the pandemic.

Our age‐specific admission rates are also comparable with other English studies (Bolotin et al., 2012; Campbell et al., 2011) for patients younger than 24 years old but higher for patients older than 25 years old. Variation between our and other studies' findings may be due to country differences. However, since most rely on laboratory‐confirmed data from an ungeneralizable subsample of hospitals, it is likely that they also fail to capture a substantial proportion of adult H1N1 admissions and therefore underreport pandemic influenza's burden on their hospitals. To correct for the underreporting of previous studies due to data limitations, we use our findings to adjust the estimates reported for the United States (Shrestha et al., 2011), Spain (Galante et al., 2012), Greece (Zarogoulidis et al., 2012), Australia (Higgins et al., 2011), and New Zealand (Wilson et al., 2012) by multiplying them by 1.3 as a lower bound and 4.3 as an upper bound to achieve corrected H1N1 admissions estimates (Table 4).

**Table 4:** Correction of previous estimates of H1N1 admissions and costs based on our seasonal autoregressive integrated moving average (SARIMA)–generated findings

Country	Number of H1N1 admissions during the pandemic period (previous studies)	Number of H1N1 admissions during the pandemic period (corrected based on our estimated excess admissions)^a^ [95% CI]		H1N1 unit cost (previous studies)	Total cost of all H1N1 hospital admissions during the pandemic period(corrected based on our estimated excess admissions)^f^ [95% CI]	
		Lower bound (1.31)^b^	Upper bound (4.28)^c^		Lower bound (1.31)^b^	Upper bound (4.28)^c^
United States	274,304 (Shrestha et al., 2011)	131,145.34^d^ [119,132.03, 144,159.77]	428,474.87 [388,430.49, 468,519.25]	$11,536.59	$1,512,970,018 [$1,374,377,385.98, $1,663,112,160.98]	$4,943,138,900.49 [$4,481,163,306.63, $5,405,114,494.36]
Spain	3,025 (Galante et al., 2012)	3,962.75 [3,599.75, 4,356]	12,947 [11,737, 14,157]	$6,603.17	$26,166,711.92 [$23,769,761.21, $28,763,408.52]	$90,578,324.16 [$77,501,406.29, $93,481,077.69]
Greece	133 (Zarogoulidis et al., 2012)	18,177.99^e^ [16,512.83, 19,981.92]	59,390.69 [53,840.16, 64,941.22]	$4,086.04	$74,276,021.50 [$67,472,094.93, $81,646,904.78]	$242,672,744.77 [$219,993,049, $265,352,440.55]
Australia and New Zealand	762 (Higgins et al., 2011)	998.22 [906.78, 1,097.28]	3,261.36 [2,956.56, 3,566.16]	$5,735.70	$5,725,490.45 [$5,201,018.05, $6,293,668.90]	$18,706,182.55 [$16,957,941.19, $20,454,423.91]
New Zealand	1,122 (Wilson et al., 2012)	1,469.82 [1,335.18, 1,615.68]	4,802.16 [4,353.36, 5,250.96]	$11,646.18	$17,117,788.29 [$15,549,746.61, $18,816,500.10]	$55,926,819.75 [$50,700,014.16, $61,153,625.33]

a Number of excess admissions had these studies used SARIMA, calculated as the number of H1N1 admissions found in each study multiplied by the range of which SARIMA estimates exceeded England studies' estimates.

b Lower bound of the range that SARIMA estimates exceeded England studies' estimates = 1.31 (95% CI: 1.19, 1.44; Bolotin et al., 2012).

c Upper bound of the range that SARIMA estimates exceeded England studies' estimates = 4.28 (95% CI: 3.88, 4.68; Campbell et al., 2011).

d Shrestha calculations were made by first dividing 274,304, their hospitalization estimates, by their corrector, 2.74, to equal 100,110.95, then multiplying by the range of which SARIMA estimates exceeded England studies' estimates.

e Zarogouldis et al. reported 133 H1N1 admissions in three hospitals; as there are 313 hospitals in Greece (European Hospital and Healthcare Federation, 2015), we calculated (313/3) × 133 = 13,876.33 H1N1 admissions across Greece. This was then multiplied by the range of which SARIMA estimates exceeded England studies' estimates.

f Excess hospitalization costs calculated as each study's country‐specific number of admissions multiplied by the corrector had SARIMA been used (columns 3 and 4) multiplied by the unit cost reported in each study (column 5).

Our estimated H1N1 unit costs are similar to the previous literature (Galante et al., 2012; Higgins et al., 2011; Uscher‐Pines & Elixhauser, 2013; Wilson et al., 2012; Zarogoulidis et al., 2012). Therefore, when combined with previous conservative admission estimates, this would suggest that actual costs may be substantially higher than what was assessed. Thus, our improved admission estimates are multiplied by their reported unit costs, resulting in country‐specific adjusted H1N1 admissions cost estimates (Table 4). Since these are back‐of‐the‐envelope calculations, they should be interpreted cautiously for two reasons. First, the algorithms for ordering laboratory tests and admitting patients are likely to differ between the United Kingdom and other countries. Second, it is possible that we still underestimate the number and cost of H1N1 admissions even after we multiply other countries' estimates by our adjuster. This is because some patients who would normally be admitted for seasonal influenza may have caught H1N1 instead. Thus, there may have been an abnormally low number of nonpandemic, seasonal influenza patients, and a higher number of H1N1 patients, during the pandemic and postpandemic periods. Therefore, these calculations improve on current studies from other countries with a greater uncertainty than those for England. Nevertheless, they are likely to closer reflect the true burden of the pandemic in these countries.

## 5 CONCLUSION

This paper characterizes the excess number and cost of ILI hospital admissions above seasonal influenza ascribed to H1N1 during the pandemic and postpandemic influenza season in England. We contribute to the literature by providing the first unit cost estimates based on individual‐level data from all hospitals during the pandemic and postpandemic period. We exploit the universe of inpatient data in England as opposed to laboratory‐confirmed admissions from a nonrandom subsample of hospitals, which may have biased previous studies' estimates.

During the pandemic, the elderly had the lowest H1N1 admission rates but the highest unit cost. Despite a significant excess number of ILI admissions, excess, all‐cause winter mortality levels remained similar to nonpandemic years (Pebody et al., 2010). We speculate that unlike a typical nonpandemic year, the majority of excess ILI hospitalizations in the pandemic composed of younger individuals with few comorbidities and low likelihood of mortality, unlike the elderly (Thompson et al., 2003). Therefore, increased ILI admissions may not have translated into increased (all‐cause) winter deaths in 2009/2010 if patients were of low complexity.

During the postpandemic period, we found increased admission rates among all age groups except 5‐ to 14‐year‐olds and increased excess unit costs compared with the pandemic among 0‐ to 4‐year‐olds and 25+‐year‐olds.

Our analysis has limitations. First, SARIMA is unable to capture any unobservable factors concurrent but not in response to the pandemic as it uses past observations to predict future values. Government actions unaffected by the pandemic, such as the scheduled opening of new hospitals, may have independently impacted the number and cost of H1N1 admissions. Second, SARIMA requires stationary data that is constant over time and therefore may not be a suitable technique for other countries due to country‐specific externalities. Third, some studies caution that ARIMA may underestimate the burden of respiratory virus‐attributable hospitalizations (Gilca, De Serres, Skowronski, Boivin, & Buckeridge, 2009). Gilca et al. (2009), however, use a laboratory‐confirmed prospective study of 6‐ to 23‐month‐olds from one hospital in one Canadian province, which may not generalize to other age groups, all of Canada or the United Kingdom. Moreover, our SARIMA models adjust for ILI seasonal fluctuations, unlike ARIMA.

Fourth, it is possible that influenza admissions were particularly low in our prepandemic data and that incorporating pre‐2004 data would strengthen our counterfactual of typical ILI admissions and costs. Previous studies, however, find similar trends and magnitude of influenza admissions to this study for earlier years (2000–2005; Elliot, Cross, & Fleming, 2008). Furthermore, other respiratory pathogens such as respiratory syncytial virus (RSV) and pneumonia may substantially contribute to the excess ILI admissions and costs during the pandemic and postpandemic period. Studies show, however, that RSV‐associated hospitalizations from 2007 to 2012, including both pandemic and postpandemic periods, have not deviated from the typical levels (Reeves et al., 2017). Likewise, 2009 featured a pandemic‐attributed spike in pneumonia incidence followed by a return to typical prepandemic levels in 2010 (British Lung Foundation, 2018). Thus, we expect the burden of other respiratory pathogens to be seasonally stable over time and captured in our counterfactual based on stationary prepandemic data. Additionally, as almost all influenza admissions during the pandemic were due to H1N1 (Bolotin et al., 2012), it is not likely that random nonpandemic‐related fluctuations in other respiratory pathogen‐related hospitalizations existed and contributed to excess ILI admissions and costs.

Furthermore, our reliance on diagnosis codes to identify ILI patients is subject to physicians' discretion unlike laboratory tests, potentially leading to miscoding and biased estimates. In principle, laboratory‐confirmed H1N1 admissions would be ideal, but in practice, they represent a small fraction of actual hospitalizations (Reed et al., 2009; Wang et al., 2010). For example, McLean and Pebody (2010) found 2,831 laboratory‐confirmed H1N1 hospitalizations (July 2009–April 2010) compared with the 10,348 excess admissions we found (June 2009–March 2010). Finally, the omission of patients who could not be matched with a cost (2.6% of data) due to data limitations may result in underestimated excess unit costs. We find that the unmatched are significantly older and have longer lengths of stay than matched admissions. Thus, we are possibly overconservative in our estimates of excess ILI cost and bias downward the costliest age group. In summary, although diagnosis codes provide us with a clear advantage over laboratory‐confirmed data sets, our data set still poses some limitations that may bias our estimates. Lastly, SARIMA models can establish a counterfactual of nonpandemic admission numbers and costs but cannot identify the causal impact of the H1N1 pandemic on hospitals.

Notwithstanding these limitations, our overall and age‐specific estimates of H1N1 hospitalizations and costs can further our understanding of secondary care use during an influenza pandemic and its aftermath. These results support the need for improved pandemic preparedness, especially strategies to cope with unexpected surges in secondary care demand. Moreover, our confirmation of a larger wave of excess ILI admissions and costs during the postpandemic influenza season suggests that authorities need to plan for severe pandemic aftershocks to health systems. Our results demonstrate that there may be value in monitoring daily or weekly ILI hospital admissions during and after pandemics. Such analyses may complement other more commonly used surveillance methods. Our unit cost estimates in particular can help inform the cost‐effectiveness of public health interventions during pandemics. Policymakers can use SARIMA on hospital administrative data sets to generate country‐specific or even global estimates that will aid their own future pandemic preparedness planning (Abrahams et al., 2013; United States Homeland Security Council, 2006).

## CONFLICT OF INTEREST

We declare that we have no conflicts of interest.

## AUTHOR CONTRIBUTIONS

K. L. undertook the estimation, wrote most of the paper, and contributed to the research idea and preparation of the data. K. H. and M. M. conceived the research idea, prepared the data, and contributed to the analysis and writing of the paper.

## Supporting information

Table S1 Previous vs. our estimates of overall and highest age‐group's weekly H1N1 hospital admission rate during the pandemic and post‐pandemic period
Table S2 Previous estimates of unit costs for H1N1 hospital admissions
Table S3 SARIMA (p,d,q) (P,D,Q)_m_^a^ models used to determine overall and age group specific excess admissions and costs attributed to H1N1 for the pandemic and post‐pandemic period: June 2009 – March 2011
Table S4 Parameter estimates of SARIMA (p,d,q) (P,D,Q)_m_ models and Ljung‐Box and Bartlett test statistics for overall and age‐split excess hospital admissions attributed to H1N1
Table S5 Parameter estimates of SARIMA (p,d,q) (P,D,Q)_m_ models and Ljung‐Box and Bartlett test statistics for overall and age group specific excess costs attributed to H1N1
