# Supplementary material for: Excess influenza hospital admissions and costs due to the 2009 H1N1 pandemic in England
Source: Health Econ. 2018 Oct 18;28(2):175–88. doi: 10.1002/hec.3834 (PMC6491983; doi:10.1002/hec.3834)
Supplement: Supplementary file 1 — Table S1 Previous vs. our estimates of overall and highest age‐group's weekly H1N1 hospital admission rate during the pandemic and post‐pandemic period Table S2 Previous estimates of unit costs for H1N1 hospital admissions Table S3 SARIMA (p,d,q) (P,D,Q)m a models used to determine overall and age group specific excess admissions and costs attributed to H1N1 for the pandemic and post‐pandemic period: June 2009 – March 2011 Table S4 Parameter estimates of SARIMA (p,d,q) (P,D,Q)m models and Ljung‐Box and Bartlett test statistics for overall and age‐split excess hospital admissions attributed to H1N1 Table S5 Parameter estimates of SARIMA (p,d,q) (P,D,Q)m models and Ljung‐Box and Bartlett test statistics for overall and age group specific excess costs attributed to H1N1 [file HEC-28-175-s001.docx]

Supplementary Appendix

**Table S1** Previous vs. our estimates of overall and highest age-group’s weekly H1N1 hospital admission rate during the pandemic and post-pandemic period

| **Author** | **Country** | **Type of Admissions Data ^a^** | **Calculation Approach** | **Dates of Pandemic**  **(no. of weeks)** | | **Dates of Post-Pandemic**  **(no. of weeks)** | **Pandemic Weekly H1N1 Admission Rate ^c^** | | **Post –Pandemic Period Weekly H1N1 Admission Rate ^c^** | |
| --- | --- | --- | --- | --- | --- | --- | --- | --- | --- | --- |
|  |  |  |  |  |  |  | **Overall** | **Highest Age Group’s Rate** | **Overall** | **Highest Age Group’s Rate** |
| Campbell et al. (2011) | England | Laboratory-confirmed H1N1 from a surveillance system of 77% of all hospitals | Counting | April 1, 2009 –  January 6, 2010  (42 weeks) | | NA | 0.11 | 0.66  (0-1 years) | NA | NA |
| Mytton et al. (2012) | England | All influenza (seasonal and H1N1) admissions in a patient-level administrative dataset | Counting | June 29, 2009 –  February 28, 2010  (35 weeks) | | October 4, 2010 –  February 27, 2011  (21 weeks) | 0.43 | NA | 0.79 | NA |
| Bolotin et al. (2012)  ^a^ | England | Laboratory-confirmed H1N1 from a sentinel network of 13.7% of all hospitals | Counting | April 1, 2009 –  January 6, 2010  (42 weeks) | | October 4, 2010 –  February 13, 2011  (19 weeks) | 0.17 ^b^ | 0.06  (0-4 years) | 0.91 ^b^ | 0.28  (0-4 years) |
| Presanis et al. (2011) | England | Laboratory-confirmed H1N1 from a surveillance system of 77% of all hospitals | Bayesian modelling | June 1 – August 31, 2009 (13 weeks) &  September 1, 2009 – February 28, 2010 (26 weeks) | | NA | 0.48 &  0.56  Total:  0.53 | NA | NA | NA |
| Shrestha et al. (2011) | United States | Laboratory-confirmed H1N1 from a surveillance system covering 60 counties in 12 metropolitan areas in 10 states | Hospitalization rates with correction for under-reporting | April 12, 2009 –  April 10, 2010  (52 weeks) | | NA | 1.72 | 2.26  (0-17 years) | NA | NA |
| Helferty et al. (2010) | Canada | Laboratory-confirmed H1N1 from a surveillance system covering all provinces/territories | Counting | April 18, 2009 –  April 3, 2010  (50 weeks) | | NA | 0.43 | 3.31  (0-1 years) | NA | NA |
| Van’t Klooster et al. (2010) | Netherlands | Laboratory-confirmed H1N1 from a nationwide mandatory notification system | Counting | June 5, 2009 –  December 31, 2009  (30 weeks) | | NA | 0.45 | 2.09  (0-5 years) | NA | NA |
| This study’s SARIMA-generated estimates | England | All ILI (seasonal and H1N1) admissions in a patient-level administrative dataset | SARIMA to disentangle excess over seasonal influenza attributed to H1N1 | June 8, 2009 – March 7, 2010  (38 weeks) | November 22, 2010 – March 27, 2011  (18 weeks) | | 0.52 | 0.11  (0-4 years) | 1.23 | 0.20  (0-4 years) |

^a^ Data was collected in 2009/10 for the pandemic and 2010/11 for the post-pandemic influenza season

^b^ Bolotin et al. (2012) reported 409 laboratory-confirmed H1N1 hospitalizations for 11.3% of all NHS hospitals in the pandemic and 1,260 hospitalizations for 13.7% of hospitals in the post-pandemic period.. When scaled to a national level, we estimate 3,619 and 9,197 H1N1 admissions, respectively. These informed the weekly H1N1 admission rate calculations.

^c^ Weekly H1N1 admission rate calculated as [(number of H1N1 hospital admissions / number of weeks in pandemic or post-pandemic period) / population of that country in 2009 (for the pandemic) or 2010 (for the post-pandemic period)] * 100,000

**Table S2** Previous estimates of unit costs for H1N1 hospital admissions

| **Author** | **Country** | **Type of Costing Data ^a^** | **Type of Admissions Data used to Calculate Unit Costs** | **Calculation Approach** | **Unit Cost ^b^** |
| --- | --- | --- | --- | --- | --- |
| Uscher-Pines and Elixhauser (2013) | United States | Survey data of covering a 20% stratified random sample of all hospitals, excluding rehabilitation and long-term acute care hospitals | Inpatient stays for influenza (seasonal and H1N1) | Summing cost per patient reported in survey data | $11,536.59 |
| Galante et al. (2012) | Spain | 1 hospital | Laboratory-confirmed H1N1 | Bottom up  (micro-costing) | $6,603.17 |
| Zarogouldis et al. (2012) | Greece | 3 tertiary care hospitals | Laboratory-confirmed H1N1 | Bottom up  (micro-costing) | $4,086.04 |
| Higgins et al. (2011) | Australia and New Zealand | 1 hospital | Laboratory-confirmed H1N1 | Bottom up  (micro-costing) | $5,735.70 |
| Wilson et al. (2012) | New Zealand | 1 hospital | Laboratory-confirmed H1N1 | Bottom up  (micro-costing) +  gamma distribution | $11,646.18 |
| This study’s SARIMA-generated estimates | England | National tariffs, which assign a cost to every ILI admission (seasonal and H1N1) in the administrative dataset | All influenza (seasonal and H1N1) admissions in a patient-level administrative dataset | SARIMA to disentangle excess weekly costs attributable to H1N1 over seasonal influenza divided by weekly number of excess ILI patients previously determined | $3,991.72  (pandemic)  $7,684.86  (post-pandemic period) |
| ^a^ Data collected in 2009/10 | | | | | |
| ^b^ Adjusted for inflation in the originating country and converted to 2011 US dollar | | | | | |

**Table S3** SARIMA (p,d,q) (P,D,Q)_m_ ^a^ models used to determine overall and age group specific excess admissions and costs attributed to H1N1 for the pandemic and post-pandemic period: June 2009 – March 2011

| **H1N1 Admissions Category** | **Admissions** | | **Costs** | |
| --- | --- | --- | --- | --- |
|  | **Pandemic** | **Post-Pandemic** | **Pandemic** | **Post-Pandemic** |
| Overall Admissions | ARIMA*(2,0,0) (0,1,0)_52_* | ARIMA*(2,0,0) (0,1,0)_52_* | ARIMA*(1,0,1) (0,1,0)_52_* | ARIMA*(1,0,1) (0,1,0)_52_* |
| 0-4 years | ARIMA*(1,0,1) (0,1,0)_52_* | ARIMA*(1,0,1) (0,1,0)_52_* | ARIMA*(5,0,0) (0,1,0)_52_* | ARIMA*(4,0,0) (0,1,0)_52_* |
| 5-14 years | ARIMA*(1,0,2) (0,1,0)_52_* | ARIMA*(1,0,2) (0,1,0)_52_* | ARIMA*(1,0,2) (0,1,0)_52_* | ARIMA*(2,0,1) (0,1,0)_52_* |
| 15-24 years | ARIMA*(2,0,0) (0,1,0)_52_* | ARIMA*(2,0,0) (0,1,0)_52_* | ARIMA*(2,0,2) (0,1,0)_52_* | ARIMA*(1,0,2) (0,1,0)_52_* |
| 25-44 years | ARIMA*(1,0,0) (0,1,0)_52_* | ARIMA*(1,0,0) (0,1,0)_52_* | ARIMA*(2,0,2) (0,1,0)_52_* | ARIMA*(1,0,2) (0,1,0)_52_* |
| 45-64 years | ARIMA*(1,0,0) (0,1,0)_52_* | ARIMA*(1,0,0) (0,1,0)_52_* | ARIMA*(1,0,2) (0,1,0)_52_* | ARIMA*(1,0,0) (0,1,0)_52_* |
| 65+ years | ARIMA*(2,0,0) (0,1,0)_52_* | ARIMA*(2,0,0) (0,1,0)_52_* | ARIMA*(1,0,4) (0,1,0)_52_* | ARIMA*(1,0,1) (0,1,0)_52_* |
| ^a^ The SARIMA (p,d,q) (P,D,Q)_m_ model includes non-seasonal (p,d,q) and seasonal (P,D,Q) autoregressive, differencing, and moving average parameters while m refers to the number of periods in each season | | | | |

**Table S4** Parameter estimates of SARIMA (p,d,q) (P,D,Q)_m_ models and Ljung-Box and Bartlett test statistics for overall and age-split excess hospital admissions attributed to H1N1

|  | **Total Admissions** | **0-4 years** | **5-14 years** | **15-24 years** | **25-44 years** | **45-64 years** | **65+ years** | |
| --- | --- | --- | --- | --- | --- | --- | --- | --- |
| **2009** | **(1)** | **(2)** | **(3)** | **(4)** | **(5)** | **(6)** | **(7)** | |
|  | **AR(2)** | **AR(1) MA(1)** | **AR(1) MA(2)** | **AR(2)** | **AR(1)** | **AR(1)** | **AR(2)** | |
| AR Lag 1 | 0.532*** | 0.820*** | 0.493*** | 0.350*** | 0.376*** | 0.269*** | 0.200*** | |
|  | (0.057) | (0.074) | (0.177) | (0.057) | (0.049) | (0.060) | (0.077) | |
| AR Lag 2 | 0.195** |  |  | 0.162*** |  |  | 0.328*** | |
|  | (0.083) |  |  | (0.058) |  |  | (0.066) | |
| MA Lag 1 |  | -0.406*** | -0.300* |  |  |  |  | |
|  |  | (0.120) | (0.158) |  |  |  |  | |
| MA Lag 2 |  |  | 0.227*** |  |  |  |  | |
|  |  |  | (0.088) |  |  |  |  | |
| Constant | 0.608 | 0.181 | -0.022 | -0.028 | 0.197 | 0.294 | -0.139 | |
|  | (2.246) | (0.747) | (0.225) | (0.335) | (0.418) | (0.265) | (0.447) | |
| Ljung-Box | 37.035 | 28.341 | 43.465 | 43.344 | 39.382 | 34.673 | 44.975 | |
| Bartlett | 0.739 | 0.708 | 0.798 | 0.335 | 0.498 | 0.729 | 0.271 | |
| **2010/2011** | **(8)** | **(9)** | **(10)** | **(11)** | **(12)** | **(13)** | **(14)** | |
|  | **AR(2)** | **AR(1) MA(1)** | **AR(1) MA(2)** | **AR(2)** | **AR(1)** | **AR(1)** | **AR(2)** | |
| AR Lag 1 | 0.543*** | 0.792*** | 0.545*** | 0.334*** | 0.384*** | 0.266*** | 0.231*** | |
|  | (0.046) | (0.065) | (0.173) | (0.046) | (0.042) | (0.048) | (0.063) | |
| AR Lag 2 | 0.178*** |  |  | 0.169*** |  |  | 0.269*** | |
|  | (0.065) |  |  | (0.045) |  |  | (0.056) | |
| MA Lag 1 |  | -0.372*** | -0.420*** |  |  |  |  | |
|  |  | (0.099) | (0.160) |  |  |  |  | |
| MA Lag 2 |  |  | 0.167** |  |  |  |  | |
|  |  |  | (0.065) |  |  |  |  | |
| Constant | 1.124 | 0.413 | 0.051 | 0.081 | 0.388 | 0.364 | 0.064 | |
|  | (1.690) | (0.576) | (0.174) | (0.274) | (0.346) | (0.220) | (0.339) | |
| Ljung-Box | 37.287 | 28.127 | 36.232 | 50.099 | 49.454 | 47.747 | 50.365 | |
| Bartlett | 0.797 | 0.857 | 0.475 | 0.446 | 0.941 | 0.609 | 0.520 | |
| *Notes:* *** p<0.01, ** p<0.05, * p<0.01. Standard errors are in parentheses | | | | | | | |  |

**Table S5** Parameter estimates of SARIMA (p,d,q) (P,D,Q)_m_ models and Ljung-Box and Bartlett test statistics for overall and age group specific excess costs attributed to H1N1

|  | **Total Admissions** | **0-4 years** | **5-14 years** | **15-24 years** | **25-44 years** | **45-64 years** | **65+ years** |
| --- | --- | --- | --- | --- | --- | --- | --- |
| **2009** | **(1)** | **(2)** | **(3)** | **(4)** | **(5)** | **(6)** | **(7)** |
|  | **AR(1) MA(1)** | **AR(5)** | **AR(1) MA(2)** | **AR(2) MA(2)** | **AR(2) MA(2)** | **AR(1) MA(2)** | **AR(1) MA(4)** |
| AR Lag 1 | 0.819*** | 1.619*** | 0.581*** | 0.956*** | 0.961*** | 0.680*** | 0.967*** |
|  | (0.082) | (0.049) | (0.056) | (0.113) | (0.094) | (0.058) | (0.027) |
| AR Lag 2 |  | -0.740*** |  | -0.292** | -0.287** |  |  |
|  |  | (0.090) |  | (0.122) | (0.121) |  |  |
| AR Lag 3 |  | -0.476*** |  |  |  |  |  |
|  |  | (0.112) |  |  |  |  |  |
| AR Lag 4 |  | 0.827*** |  |  |  |  |  |
|  |  | (0.114) |  |  |  |  |  |
| AR Lag 5 |  | -0.339*** |  |  |  |  |  |
|  |  | (0.065) |  |  |  |  |  |
| MA Lag 1 | -0.509*** |  | 0.310*** | 0.564*** | 0.666*** | 0.874*** | 0.534** |
|  | (0.118) |  | (0.075) | (0.111) | (0.074) | (0.061) | (0.087) |
| MA Lag 2 |  |  | 0.325*** | 0.489*** | 0.710*** | 0.577*** | 0.422*** |
|  |  |  | (0.068) | (0.084) | (0.050) | (0.068) | (0.100) |
| MA Lag 3 |  |  |  |  |  |  | -0.673*** |
|  |  |  |  |  |  |  | (0.100) |
| MA Lag 4 |  |  |  |  |  |  | -0.529*** |
|  |  |  |  |  |  |  | (0.083) |
| Constant | 2169.83 | 1499.43 | 618.493 | 361.486 | 106.395 | 261.930 | 6.755 |
|  | (2657.854) | (1172.063) | (619.221) | (431.006) | (437.690) | (468.341) | (1888.11) |
| Ljung-Box | 33.589 | 43.827 | 35.433 | 47.457 | 33.651 | 75.263 | 61.898 |
| Bartlett | 0.762 | 0.686 | 0.823 | 0.454 | 0.896 | 0.660 | 1.196 |
| **2010/2011** | **(8)** | **(9)** | **(10)** | **(11)** | **(12)** | **(13)** | **(14)** |
|  | **AR(1) MA(1)** | **AR(4)** | **AR(2) MA(1)** | **AR(1) MA(2)** | **AR(1) MA(2)** | **AR(1)** | **AR(1) MA(1)** |
| AR Lag 1 | 0.821*** | 1.151*** | -0.354*** | 0.377*** | 0.636*** | 0.832*** | 0.838*** |
|  | (0.091) | (0.034) | (0.070) | (0.049) | (0.053) | (0.030) | (0.048) |
| AR Lag 2 |  | -0.313*** | 0.619*** |  |  |  |  |
|  |  | (0.064) | (0.040) |  |  |  |  |
| AR Lag 3 |  | -0.241*** |  |  |  |  |  |
|  |  | (0.068) |  |  |  |  |  |
| AR Lag 4 |  | 0.200*** |  |  |  |  |  |
|  |  | (0.052) |  |  |  |  |  |
| MA Lag 1 | -0.601*** |  | 0.849*** | 0.457*** | 0.507*** |  | -0.119* |
|  | (0.113) |  | (0.084) | (0.040) | (0.070) |  | (0.064) |
| MA Lag 2 |  |  |  | 0.615*** | 0.452*** |  |  |
|  |  |  |  | (0.040) | (0.056) |  |  |
| Constant | 2550.036 | 1587.562 | 750.901 | 337.195 | 187.106 | 268.506 |  |
|  | (2078.702) | (964.585) | (649.977) | (298.380) | (367.803) | (459.171) |  |
| Ljung-Box | 35.689 | 42.344 | 22.143 | 43.368 | 45.048 | 62.368 | 44.181 |
| Bartlett | 0.770 | 0.618 | 0.622 | 0.699 | 0.658 | 1.112 | 0.750 |
| *Notes:* *** p<0.01, ** p<0.05, * p<0.01. Standard errors are in parentheses. | | | | | | | |
